# *Astragalus* and *Cordyceps* Derivatives in the Treatment of Aging-Related Chronic Diseases and Neurodegenerative Disorders

**DOI:** 10.3390/ijms27125273

**Published:** 2026-06-10

**Authors:** Kiran Reddy Kanubaddi, Chih-Liang Yaung, Horng-Jyh Harn, Tzyy-Wen Chiou, Shao-Xi Hsu, Ivan Wijaya, Shinn-Zong Lin, Wei Wuli

**Affiliations:** 1 Department of Physics, National Dong Hwa University, Hualien 97401, Taiwan; kiranreddy@gms.ndhu.edu.tw; 2 Department of Life Science, National Dong Hwa University, Hualien 974301, Taiwan; twchiou@gms.ndhu.edu.tw (T.-W.C.); sean933275@gmail.com (S.-X.H.); 3 Department of Healthcare Administration, Asia University, Taichung 413305, Taiwan; clyaung@asia.edu.tw; 4 Department of Pathology, Hualien Tzu Chi Hospital, Hualien 97002, Taiwan; arthewduke@gmail.com; 5 Bioinnovation Center, Buddhist Tzu Chi Medical Foundation, Hualien 97002, Taiwan; ivanwjy74@gmail.com; 6 Department of Neurology, Hualien Tzu Chi Hospital, Hualien 97002, Taiwan; 7 Department of Medical Research, Hualien Tzu Chi Hospital, Buddhist Tzu Chi Medical Foundation, Hualien 97002, Taiwan

**Keywords:** aging, traditional Chinese herbal medicine, *Astragalus membranaceus*, *Cordyceps*, multi-target therapy

## Abstract

Aging is associated with a rising burden of chronic metabolic, cardiovascular, musculoskeletal, and neurodegenerative diseases that share interconnected pathological mechanisms, including oxidative stress, chronic inflammation, mitochondrial dysfunction, metabolic imbalance, and immune dysregulation. Because these disorders arise from complex and overlapping biological disturbances, conventional single-target therapies often provide only limited benefit. In this context, traditional Chinese herbal medicines, characterized by multi-component and multi-target actions, are being re-evaluated using modern pharmacological and systems biology approaches. Among these, *Astragalus membranaceus* and *Cordyceps* species have attracted attention as representative tonic medicines with long-standing traditional use and growing biomedical relevance. Their principal bioactive constituents, including polysaccharides, saponins, flavonoids, sterols, and nucleoside derivatives such as cordycepin, exert pleiotropic effects on inflammatory signaling, redox homeostasis, mitochondrial function, metabolic regulation, and immune responses. This review summarizes current evidence on bioactive derivatives from *Astragalus* and *Cordyceps* in aging-related chronic and neurodegenerative disorders, including diabetes, cardiovascular dysfunction, osteoarthritis, cancer, Alzheimer’s disease, and Parkinson’s disease. It focuses on mechanistic findings from cellular and animal studies and critically discusses key translational challenges, such as compositional variability, poor bioavailability, lack of standardized preparation, limited clinical validation, and safety concerns related to toxicity and herb–drug interactions.

## 1. Introduction

Traditional Chinese herbal medicines have been widely used for centuries due to their long history, broad accessibility, and rich chemical diversity. Advances in modern extraction, purification, and analytical technologies now allow the isolation and characterization of complex bioactive compounds from medicinal plants according to their physicochemical properties. These developments have facilitated the identification of multiple classes of natural compounds, including polysaccharides, flavonoids, saponins, polyphenols, and nucleosides, which contribute to the pharmacological activities of herbal medicines [1,2,3,4].

Aging is accompanied by a progressive decline in cellular and organ function, leading to an increased incidence of chronic diseases and neurodegenerative disorders. Elderly individuals face elevated risks of conditions such as type 2 diabetes, cardiovascular diseases (including hypertension and atherosclerosis), osteoarthritis, osteoporosis, and neurodegenerative disorders such as Alzheimer’s disease and Parkinson’s disease. These conditions frequently coexist, and many individuals over the age of 60 present with multiple comorbidities that require combination therapies, which can complicate treatment strategies and increase the risk of drug interactions [5,6]. Importantly, these aging-related diseases share several convergent pathological mechanisms, including persistent oxidative stress, chronic inflammation, mitochondrial dysfunction, metabolic dysregulation, impaired proteostasis, and altered cellular signaling pathways. The multifactorial nature of these processes makes it difficult for single-target pharmacological agents to effectively address disease progression [7,8,9].

Accordingly, increasing attention has been directed toward therapeutic strategies that can regulate multiple interconnected pathways involved in aging-related diseases. Traditional Chinese Medicine (TCM) is especially relevant in this regard because herbal medicines typically contain multiple bioactive constituents that act on interconnected molecular and cellular networks. Unlike conventional drugs designed for a single target, herbal-derived compounds often exert pleiotropic effects across diverse biological pathways, allowing them to influence complex disease microenvironments in a context-dependent manner. Modern studies increasingly describe such medicines as “multi-component, multi-target, multi-pathway” therapeutics, and many reports suggest that they can modulate aging-related processes through coordinated effects on inflammation, oxidative injury, metabolism, and stress-response pathways [10,11,12].

Among the many herbs used in TCM, *Astragalus membranaceus* (Huangqi) and *Cordyceps* species were selected for this review because they provide a scientifically useful comparison of two distinct medicinal materials that converge on several aging-related pathological mechanisms. *Astragalus* contains polysaccharides, flavonoids, and saponins, whereas *Cordyceps* produces bioactive metabolites such as cordycepin, adenosine, polysaccharides, sterols, and peptides. Experimental studies have shown that these constituents possess antioxidant, anti-inflammatory, immunomodulatory, metabolic, and cytoprotective activities. *Astragalus* has demonstrated protective effects in models of cardiac, cerebral, renal, hepatic, and pulmonary injury, while cordycepin and related *Cordyceps*-derived compounds have shown promising neuroprotective and anti-inflammatory activities, including attenuation of mitochondrial dysfunction and inflammasome activation in Parkinson’s disease models [13,14,15,16,17]. Together, *Astragalus* and *Cordyceps* represent multi-component herbal agents with the potential to target several hallmarks of aging simultaneously.

Although *Astragalus* is a plant-derived medicine and *Cordyceps* is a fungus-derived medicine, both have been reported to regulate biological mechanisms closely associated with aging, including oxidative stress, chronic inflammation, mitochondrial dysfunction, metabolic imbalance, immune dysregulation, and neuroinflammatory injury [13,18]. Therefore, discussing these two medicines together allows a balanced comparison of how different natural product systems may influence similar aging-related pathological mechanisms.

From a traditional perspective, both herbs are regarded as restorative tonics used to improve vitality, resilience, and recovery from chronic weakness. *Astragalus* is primarily associated with qi tonification, whereas *Cordyceps* is traditionally linked to support of the lung and kidney systems. Although these traditional concepts are not directly equivalent to modern biomedical mechanisms, they provide a historical rationale for the continued investigation of these herbs in aging-related disorders. Increasingly, modern translational studies attempt to connect such traditional indications with measurable biological processes, including immune dysregulation, mitochondrial impairment, chronic inflammation, and reduced regenerative capacity [19].

This review evaluates the current literature on *Astragalus* and *Cordyceps* derivatives in aging-related chronic diseases and neurodegenerative disorders. We focus primarily on mechanistic evidence from in vitro and animal studies in models of diabetes, cardiovascular disease, osteoarthritis, cancer, Alzheimer’s disease, and Parkinson’s disease, while also considering available human studies with appropriate caution. In addition to therapeutic mechanisms, this review also considers safety profiles, dose-dependent toxicities, vulnerable patient populations, and potential herb–drug interactions. By emphasizing shared disease mechanisms and the multi-target pharmacology of these herbs, this review aims to provide an integrated and balanced overview of their therapeutic potential, current limitations, and translational prospects in modern biomedical research.

## 2. Background and Significance

### 2.1. From Traditional Chinese Medicine (TCM) to Systems-Level Pharmacology

The pharmacological interpretation of TCM differs from the reductionist paradigms that have historically dominated Western drug discovery. Conventional pharmaceutical development has largely followed the “one gene, one target, one disease” model, in which a single molecule acts on a specific molecular target to treat a defined pathology [20,21]. Although this approach has been effective for acute and well-defined conditions, it is often insufficient for multifactorial, chronic, and aging-related diseases, were pathology results from dysregulation across multiple biological pathways. In contrast, TCM formulations typically contain multiple herbs and chemically diverse constituents that collectively regulate interconnected biological processes and support physiological homeostasis [22,23]. This systems-oriented therapeutic philosophy aligns with modern views of complex disease biology and requires integrative analytical frameworks to clarify the mechanisms underlying TCM efficacy.

Polypharmacology refers to the ability of a single compound or a combination of compounds to interact with multiple biological targets and produce therapeutic effects [24]. This concept provides a mechanistic basis for TCM practice, where efficacy is often attributed to interactions among multiple constituents rather than isolated molecular actions. In aging-related disorders, including metabolic disease, cardiovascular dysfunction, and neurodegeneration, pathogenesis involves overlapping mechanisms such as oxidative stress, chronic inflammation, mitochondrial dysfunction, and immune dysregulation [22]. Therefore, multi-target modulation is particularly relevant for these complex diseases. Network pharmacology further extends this concept by integrating systems biology, computational modeling, and bioinformatics to map interactions between herbal constituents and disease-associated molecular networks [25]. This approach helps explain how complex botanical preparations influence biological networks and provides a rational framework for interpreting TCM interventions [23,26].

Beyond network-level interactions, allosteric modulation may provide additional insight into the regulatory actions of some herbal compounds. In this mechanism, ligands bind to sites distinct from the active site and induce conformational changes that fine-tune protein function [27]. Such effects may partly explain the mild and sustained physiological responses reported for certain phytochemicals, although allosteric modulation should not be viewed as a universal mechanism of TCM efficacy [28].

Another important dimension of TCM pharmacology is the interaction between orally administered herbal compounds and the gut microbiota. The gastrointestinal tract contains a complex microbial ecosystem that contributes to host metabolism, immune regulation, and inflammatory control. A bidirectional relationship exists between herbal medicines and the gut microbiome [29]. Herbal constituents such as polysaccharides and saponins can act as prebiotics, reshaping microbial composition and metabolic activity, while gut microbes can convert poorly absorbed parent compounds into bioactive metabolites with improved systemic availability [30,31]. This interaction is especially relevant to aging-related metabolic and inflammatory disorders, where microbiome dysbiosis is commonly observed. Short-chain fatty acids generated from microbial fermentation of polysaccharides can also exert immunomodulatory, anti-inflammatory, and metabolic effects. Therefore, the gut–microbiota axis represents a key interface through which herbal medicines may produce systemic effects [29,32,33,34,35].

In parallel, modernization of Chinese herbal medicine has been supported by advances in extraction, purification, and standardization technologies. These approaches help convert complex plant materials into more reproducible preparations suitable for pharmacological, mechanistic, efficacy, and safety studies [36].

Collectively, these systems-level mechanisms help explain the reported antioxidant, anti-inflammatory, metabolic, and immunomodulatory effects of Chinese herbal medicines in aging-related disorders.

### 2.2. Major Bioactive Compound Classes and Structural Considerations

**Figure 1:**
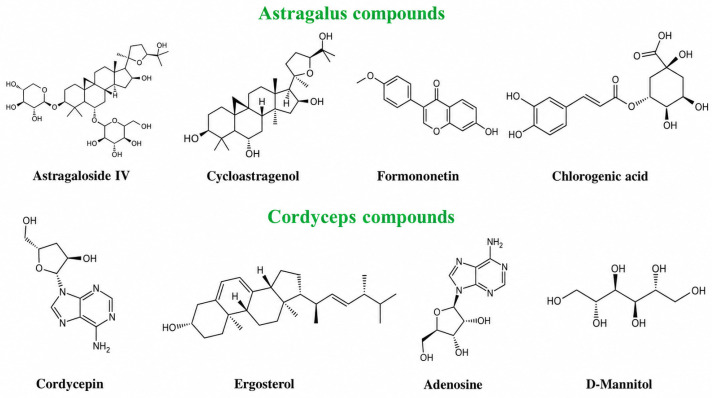
Representative bioactive compounds identified in *Astragalus* and *Cordyceps*. The upper panel shows selected *Astragalus*-derived compounds, including Astragaloside IV, Cycloastragenol, Formononetin, and Chlorogenic acid. The lower panel shows selected *Cordyceps*-derived compounds, including Cordycepin, Ergosterol, Adenosine, and D-Mannitol.

The therapeutic potential of medicinal herbs stems from a diverse array of bioactive compounds, each with distinct chemical structures that dictate their biological activities, pharmacokinetics, and mechanisms of action. Understanding the relationship between chemical structure and function is paramount for developing a rational basis for the use of herbs like *Astragalus* and *Cordyceps*. The major classes of compounds include saponins, polysaccharides, flavonoids, triterpenoids, organic acids, alkaloids, nucleosides, cyclic peptides, and sterols, representative examples of which are illustrated in Figure 1. Each class possesses characteristic structural motifs that influence its solubility, stability, interaction with biological systems, and suitability for different extraction strategies.

#### 2.2.1. Saponins

Saponins are a prominent class of glycosides widely distributed in plants and fungi, including the roots of *Astragalus* and *Panax ginseng*, and are known for their diverse bioactivities such as immunostimulant, anti-inflammatory, and anticancer properties. Structurally, they are defined by a non-sugar component called an aglycone (or genin), which can be a triterpene or a steroid, linked to one or more sugar moieties (glycones) via glycosidic bonds. The triterpene backbone, composed of six isoprene units, forms the core scaffold of many plant saponins [37,38,39]. The presence of both a hydrophobic aglycone and a hydrophilic glycone makes saponins amphiphilic molecules. This dual nature is directly responsible for their ability to interact with cell membranes, a property that underlies many of their effects, including hemolytic activity and modulation of membrane transporters [28,39]. However, this same amphiphilicity, combined with their often large molecular weight due to extensive glycosylation, contributes significantly to their poor oral bioavailability. Consequently, the systemic effects of many saponins are contingent upon their metabolism by the gut microbiota. Bacterial enzymes in the colon cleave off the sugar residues, generating smaller, more lipophilic sapogenins that are better able to cross intestinal barriers and enter systemic circulation [40,41].

#### 2.2.2. Polysaccharides

Polysaccharides represent another cornerstone of TCM pharmacology, recognized for their potent immunomodulatory and anti-inflammatory activities. They are found in high concentrations in herbs such as *Astragalus membranaceus*, *Angelica sinensis*, and *Ophiopogon japonicus* [42,43]. These compounds are polymers composed of repeating units of monosaccharides, and their structure is characterized by immense diversity in terms of molecular weight, monosaccharide composition, types of glycosidic linkages (e.g., α or β linkages), and degree of branching [37,44]. This structural variability directly correlates with their functional specificity. A critical consequence of their macromolecular structure is extremely limited oral absorption; the large size of intact polysaccharide chains prevents their passive diffusion across the intestinal epithelium [32]. Therefore, their primary mechanism of action is often indirect. Polysaccharides traverse the small intestine undigested and reach the colon, where they serve as substrates for fermentation by the resident gut microbiota [45]. This microbial degradation produces a range of bioactive metabolites, most notably short-chain fatty acids (SCFAs) like butyrate, propionate, and acetate. These SCFAs are readily absorbed and exert systemic effects by regulating immune cell function, modulating inflammatory pathways (e.g., NF-κB), and influencing host metabolism [31,34,46]. This gut-microbiota-polysaccharide axis highlights a key feature of TCM: the therapeutic effect is realized not by the parent compound itself, but by the community of metabolites it engenders.

#### 2.2.3. Flavonoids

Flavonoids are a large and structurally diverse group of polyphenolic compounds found ubiquitously in plants, contributing to color, defense, and various pharmacological activities. Their core structure consists of a C6-C3-C6 skeleton, typically comprising two aromatic rings (A and B) connected by a three-carbon bridge, which is often part of a heterocyclic ring (C) [37,47]. The biological activity and bioavailability of flavonoids are profoundly influenced by their hydroxylation and glycosylation patterns. Glycosylation generally increases water solubility but decreases lipid permeability and can protect the aglycone from oxidation [48]. Upon ingestion, flavonoids undergo extensive Phase II metabolism in the liver and intestinal cells, involving conjugation reactions catalyzed by enzymes such as UDP-glucuronosyltransferases (UGTs) and sulfotransferases (SULTs). This process adds glucuronide or sulfate groups, making the compounds more hydrophilic for excretion but often diminishing their intrinsic biological activity [49]. Furthermore, flavonoids can interact with efflux transporters like P-glycoprotein (ABCB1), which can limit their net absorption by pumping them back into the gut lumen. This complex interplay of absorption, metabolism, and transporter interactions results in low and variable oral bioavailability for most dietary flavonoids, a key consideration in translating their promising in vitro activities into clinical efficacy [48,50].

#### 2.2.4. Nucleosides

The bioactive compounds of *Cordyceps*, a fungus widely used in TCM, include a unique class of nucleoside analogues, among which cordycepin is a prominent example. Structurally, cordycepin is an adenosine analogue that lacks a hydroxyl group (–OH) at the 3′ position of its ribose sugar. Although this structural difference appears minor, it has profound pharmacological implications. The absence of the 3′-hydroxyl group makes cordycepin highly susceptible to phosphorylation by cellular kinases, resulting in its rapid conversion to cordycepin triphosphate. This active metabolite can be incorporated into RNA during transcription, leading to premature chain termination and disruption of RNA synthesis. This mechanism forms the basis of cordycepin’s antitumor and antimicrobial activities. However, the same susceptibility to phosphorylation also causes rapid intracellular metabolism and a very short half-life, which presents a major limitation for its therapeutic application [40,51,52]. This example clearly illustrates how a specific structural feature can determine a compound’s biological potency, selectivity, and pharmacokinetic profile. In addition to nucleoside analogues, other classes of compounds such as sterols, coumarins (e.g., those found in *Angelica sinensis*), and alkaloids further contribute to the chemical complexity of medicinal herbs. Many of these compounds exert their effects through the modulation of nuclear receptors and other cellular signaling pathways [38,53,54].

#### 2.2.5. Organic Acids

Organic acids contribute significantly to the bioactivity of *Astragalus* and *Cordyceps* preparations. In *Astragalus* species, the organic acid fraction is dominated by phenolic acids such as syringic, gallic, chlorogenic, caffeic, ferulic, sinapic and *p-*coumaric acids [55]. These compounds share the defining structural motif of a substituted benzene ring bearing at least one phenolic hydroxyl group and a carboxylic acid moiety; chlorogenic and ferulic acids are hydroxycinnamates with extended conjugated side chains, whereas gallic and syringic acids are hydroxybenzoates. The conjugated aromatic system and phenolic substituents afford antioxidant activity, while the carboxyl group makes these molecules weak acids. The presence of multiple hydroxyl groups and a carboxylate confers moderate polarity and water/ethanol solubility; molecular weights range from ~170 to 200 Da. At physiological pH these acids are mostly dissociated, which limits passive diffusion across lipid membranes but allows formation of salts or conjugates [56,57,58,59]. Following ingestion they undergo extensive phase II metabolism (glucuronidation, sulfation and methylation) and microbial degradation before systemic absorption; their oral bioavailability therefore tends to be low, and metabolites such as ferulic acid glucuronide predominate in plasma [60].

In *Cordyceps*, low-molecular-mass carboxylic acids and polyols are abundant. NMR-based metabolomic profiling detected citric, acetic and fumaric acids together with glucose and mannitol (often termed cordycepic acid) in extracts of *Cordyceps sinensis* [61]. Citric acid has a tricarboxylic structure (C_6_H_8_O_7_) that is highly polar and fully ionised at physiological pH, whereas fumaric and acetic acids are small (C_4_H_4_O_4_ and C_2_H_4_O_2_), dicarboxylic and monocarboxylic acids. These molecules are highly water-soluble and enter central metabolic pathways (e.g., the tricarboxylic acid cycle); they have high oral bioavailability and are rapidly metabolised to carbon dioxide and water. D-mannitol (C_6_H_14_O_6_) is a six-carbon sugar alcohol with six hydroxyl groups and no carboxyl function. Although traditionally referred to as cordycepic acid, it is a neutral polyol that functions as a carbohydrate reserve for the fungus [62]. Mannitol is strongly hydrophilic, has a moderate molecular weight (~182 Da) and does not ionise. Its hydrophilicity allows easy extraction in hot water, but its lack of lipophilicity restricts membrane permeation and it is largely excreted unchanged in urine [63,64]. Together, these acids and polyols contribute to the osmotic activity and metabolic benefits of *Cordyceps* preparations.

#### 2.2.6. Alkaloids

Compared with saponins and polysaccharides, alkaloids are less abundant in *Astragalus* and *Cordyceps* but impart distinctive pharmacological activities, such as anticancer and neuroprotective effects. Alkaloids are nitrogenous organic compounds that are typically heterocyclic and basic. Their basicity allows them to form salts with acids and exist in protonated or unprotonated forms depending on pH, which influences solubility and membrane permeation [62,65,66].

In *Astragalus* species, indolizidine and quinolizidine alkaloids have been reported. A comprehensive review of A. hamiensis found that this species contains the indolizidine alkaloid swainsonine and small amounts of the quinolizidine alkaloids ermopsine and anagyrine [67]. Indolizidines and quinolizidines are bicyclic systems derived from lysine; swainsonine contains a hydroxylated indolizidine ring that renders it moderately polar and allows hydrogen bonding, whereas ermopsine and anagyrine possess a quinolizidine skeleton that is more hydrophobic and tertiary amine functionality that imparts basicity. These alkaloids are uncharged at high pH but form cations in acidic media, which enhances solubility in polar solvents. Their lipophilicity allows passive diffusion through biological membranes; after absorption they are subject to hepatic oxidative metabolism and renal excretion. Swainsonine is a known α-mannosidase inhibitor and exhibits immunomodulatory and neurotoxic effects; bioavailability in humans has not been systematically studied, but animal models indicate good oral absorption and rapid distribution [67,68].

In *Cordyceps*, nucleoside-type alkaloids dominate. Cordycepin (3′-deoxyadenosine) comprises an adenine base linked to a ribose lacking the 3′-hydroxyl group; its structure closely resembles adenosine but the missing hydroxyl confers metabolic stability and allows incorporation into RNA, causing chain termination. N6-hydroxyethyl adenosine (HEA) has a hydroxyethyl group on the exocyclic nitrogen of the adenine ring [69]. These nucleoside analogues are neutral at physiological pH, highly hydrophilic due to the ribose and hydroxyethyl substituent and have moderate molecular weights (~267 Da); they are absorbed via nucleoside transporters and cross the blood–brain barrier. However, they are subject to rapid deamination by adenosine deaminase, reducing oral bioavailability; chemical or pharmaceutical modification (e.g., co-administration with deaminase inhibitors) is often required to enhance stability. Another group of *Cordyceps* alkaloids are militarinones, which consist of pyridine or tetramic acid (pyrrolidine-2,4-dione) cores appended to unsaturated side chains [62].

These molecules are moderately lipophilic and contain lactam or aromatic nitrogen atoms; they exist predominantly in the neutral form at physiological pH and can cross lipid membranes. Militarinones exhibit antimicrobial and cytotoxic activities and are susceptible to oxidative metabolism. Together, nucleoside and non-nucleoside alkaloids from *Cordyceps* provide diverse physicochemical profiles that influence their pharmacokinetics and therapeutic potential.

Many Astragalus and Cordyceps constituents regulate oxidative stress, inflammatory signaling, mitochondrial function, and apoptosis in cellular and animal models; however, their in vivo relevance depends on absorption, metabolic transformation, tissue distribution, and achievable systemic concentrations [70]. High-molecular-weight polysaccharides generally show poor direct intestinal absorption and may act mainly through gut microbiota-derived metabolites, short-chain fatty acid production, and gut–immune interactions rather than direct systemic exposure [71]. Similarly, saponins such as AS-IV may show limited oral bioavailability because of poor membrane permeability, intestinal metabolism, and microbial deglycosylation [72], while flavonoids are often extensively metabolized by glucuronidation, sulfation, methylation, and efflux transporter activity [73]. Cordyceps nucleoside derivatives, including cordycepin, may also undergo rapid enzymatic metabolism, which can reduce systemic persistence and alter tissue exposure [74]. Therefore, pathway-level findings should not be directly extrapolated to clinical efficacy without considering oral bioavailability, active metabolites, dose, formulation strategy, plasma concentration, tissue distribution, and target-organ exposure.

### 2.3. Extraction and Standardization of Bioactive Compounds

The extraction of bioactive compounds from *Astragalus membranaceus* and *Cordyceps* species depends primarily on the polarity, molecular size, and thermal stability of the target constituents [75]. Hydrophilic compounds such as polysaccharides and certain organic acids are generally soluble in polar solvents, whereas more lipophilic molecules, including some alkaloids and sterol derivatives, require less polar solvents for efficient extraction [76]. Therefore, the choice of extraction strategy must consider the physicochemical properties of the target compounds in order to preserve their structural integrity and biological activity.

**Figure 2:**
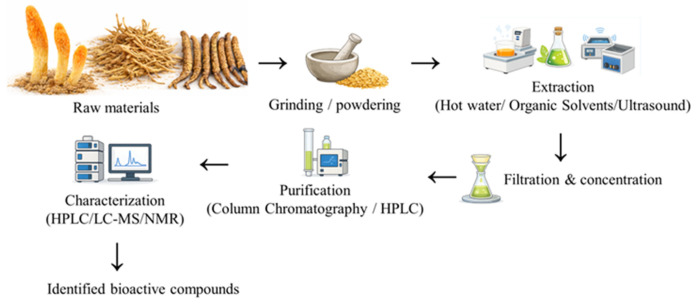
Workflow illustrating the extraction, purification, and chemical characterization of bioactive compounds from *Astragalus* and *Cordyceps*. Some icons were created with BioRender.com.

A general workflow for the extraction, purification, and analytical characterization of *Astragalus* and *Cordyceps* bioactive compounds is illustrated in Figure 2.

Traditional hot-water decoction remains one of the most widely used extraction methods in Chinese herbal medicine. Boiling water efficiently extracts polar constituents such as polysaccharides, organic acids, and certain nucleoside derivatives from *Astragalus* and *Cordyceps*. For example, water extraction of *Astragalus* roots at elevated temperatures can yield significant amounts of *Astragalus* polysaccharides, although other compounds such as flavonoids and saponins may also be co-extracted [77]. Because some phenolic compounds are sensitive to prolonged heating, careful control of extraction time and temperature is necessary to minimize degradation.

Hydroalcoholic solvent extraction using aqueous ethanol or methanol (typically 50–70%) is commonly applied to isolate moderately polar compounds such as flavonoids, phenolic acids, and nucleoside derivatives including cordycepin. Compared with water extraction, hydroalcoholic solvents often improve extraction efficiency while reducing thermal degradation of sensitive compounds. In some cases, mild acidification of the solvent can enhance the recovery of basic alkaloids by promoting protonation and improving solubility [78,79].

In recent years, several assisted extraction techniques have been explored to improve extraction efficiency and reduce processing time. Ultrasound-assisted extraction (UAE), for example, enhances mass transfer through cavitation and disruption of plant or fungal cell structures, thereby increasing the recovery of bioactive molecules. Studies on *Cordyceps militaris* have reported improved cordycepin yield when ultrasound is combined with hydroalcoholic solvents under moderate temperatures [80]. Other approaches, such as microwave-assisted extraction or enzyme-assisted extraction, may also enhance the release of intracellular compounds while maintaining relatively mild extraction conditions [77,81].

More advanced techniques, including pulsed electric field treatment, supercritical carbon dioxide extraction, and pressurized hot-water extraction, have also been investigated for isolating specific compound classes. These methods may improve extraction efficiency for certain lipophilic or thermolabile constituents [82]; however, their application to *Astragalus* and *Cordyceps* remains relatively limited compared with conventional solvent-based extraction approaches.

Overall, the extraction strategy for *Astragalus* and *Cordyceps* derivatives must balance extraction efficiency with preservation of compound stability. Optimization of solvent polarity, temperature, and extraction conditions is essential for obtaining standardized extracts enriched in key bioactive compounds while maintaining their pharmacological activity.

The major classes of bioactive compounds present in *Astragalus* and *Cordyceps*, together with their structural characteristics, biological roles, and typical extraction techniques, are summarized in Table 1.

### 2.4. Bioactive Constituents and Pharmacological Profiles of Astragalus and Cordyceps

*Astragalus membranaceus* and *Cordyceps* species are traditional tonic medicines with distinct biological origins and chemical compositions. *Astragalus* is a plant-derived medicine mainly characterized by polysaccharides, triterpenoid saponins such as AS-IV, and flavonoids, whereas *Cordyceps* is a fungus-derived medicine containing cordycepin, adenosine-related nucleosides, polysaccharides, sterols, and other fungal metabolites. Despite these differences, both have been reported to exert pharmacological effects relevant to aging-associated chronic diseases and neurodegenerative disorders, including antioxidant, anti-inflammatory, immunomodulatory, mitochondrial-protective, anti-apoptotic, and neuroprotective activities. Therefore, these two medicines should not be regarded as chemically identical or interchangeable, but as useful comparative examples of distinct natural product systems that converge on overlapping aging-related disease processes [87,90,91,92,93].

The rationale for discussing them together is not based only on their traditional classification as tonic medicines, but also on their convergence in aging-associated disease contexts, including immune dysregulation, oxidative injury, chronic inflammation, metabolic imbalance, mitochondrial decline, tissue degeneration, and neuroinflammatory damage [9,90]. At the same time, their strengths are complementary. Astragalus has been more widely studied in immune regulation, cardiometabolic protection, and adjuvant cancer therapy, whereas Cordyceps has stronger relevance to energy metabolism, fatigue-related conditions, renal protection, mitochondrial function, and neuroimmune regulation [87,92,94]. Thus, their comparative evaluation allows a balanced assessment of two distinct plant- and fungus-derived medicinal systems across shared aging-related disease areas, while also highlighting differences in active constituents, pharmacokinetics, safety concerns, evidence strength, and translational readiness.

#### 2.4.1. Cordyceps (*Ophiocordyceps sinensis*)

*Ophiocordyceps sinensis*, a parasitic fungus often referred to as *Cordyceps*, has a long history as a restorative tonic traditionally used to support vitality and stamina. Chemical investigations reveal a diverse array of secondary metabolites, including nucleosides (adenosine, inosine, guanosine, uridine and the unique 3′-deoxyadenosine cordycepin), sterols (ergosterol, ergosterol peroxide and campesterol), cyclic peptides, polyketides, phenolics and a variety of polysaccharides [95]. Cordycepin and D-mannitol, historically referred to as cordycepic acid, are prominent marker compounds; cordycepin differs from adenosine by lacking a 3′-hydroxyl group, enabling it to interfere with nucleic acid synthesis and activate adenosine receptors. *Cordyceps* polysaccharides, comprising glucans, mannans and heteroglycans, constitute 3–8% of the fruiting body and exhibit hypoglycemic, antitumor, immunomodulatory and antioxidant activities. The fungus also contains rare cyclic dipeptides, polyamines and unsaturated fatty acids with potential bioactivity [96].

*Cordycepin* may undergo rapid enzymatic metabolism; therefore, its reported effects on oxidative stress, inflammation, and mitochondrial regulation should be interpreted as exposure-dependent mechanisms rather than direct evidence of sustained systemic activity [97,98].

Traditional usage attributes *Cordyceps* with supporting vitality and respiratory function; modern pre-clinical studies support these claims by showing that *C. militaris* extracts promote neurite outgrowth, reverse scopolamine-induced memory deficits, reduce inflammatory markers (COX-2, iNOS) and oxidative stress, and upregulate dopaminergic pathways [62,99]. Polysaccharide-rich fractions lower blood glucose, suppress tumor growth and synergize with chemotherapeutic agents. In a rat model of ischemic stroke, butanolic *Cordyceps* extract decreased inflammatory cell infiltration and preserved the blood-spinal cord barrier, reflecting its anti-inflammatory and neuroprotective potential. Nevertheless, certain *Cordyceps* species produce immunosuppressive agents such as cyclosporine, and the concentrations of nucleosides and polysaccharides differ between wild *C. sinensis* and cultivated *C. militaris* [100,101,102].

#### 2.4.2. Astragalus (*Astragalus membranaceus*)

*Astragalus membranaceus* (Huangqi) is a Qi-tonifying leguminous herb traditionally used to strengthen the spleen and lungs. Chemical analyses have identified more than 200 constituents, with major classes including water-soluble heteropolysaccharides (*Astragalus* polysaccharides, APS), triterpenoid saponins (astragalosides), flavonoids and other phenolics [18]. APS are complex polysaccharides composed of glucans and acidic heteropolysaccharides; they modulate immune cell activity, regulate PI3K/Akt signaling and suppress inflammatory mediator release.

AS-IV, a cycloartane-type triterpenoid saponin isolated from *Astragalus membranaceus*, is the most extensively studied marker compound; it exerts anti-inflammatory and neuroprotective effects by suppressing oxidative stress, apoptosis, and neuroinflammation through modulation of multiple signalling pathways, including AMPK/mTOR, PPARγ/BDNF, and inflammatory cascades such as NF-κB-related responses [103,104,105,106]. Cycloastragenol, the aglycone metabolite of AS-IV, exhibits similar biological activities, including attenuation of inflammation and regulation of lipid metabolism, supporting its role as a bioactive derivative of AS-IV. Flavonoids present in *A. membranaceus*, such as quercetin and isorhamnetin, further contribute antioxidant and anti-inflammatory effects, complementing the pharmacological profile of the plant [94,107]. Because *Astragalus* polysaccharides, saponins, and flavonoids differ markedly in absorption and metabolism, their regulation of Nrf2, NF-κB, AMPK/mTOR, PI3K/Akt, and apoptosis-related pathways may reflect both parent compounds and bioactive metabolites [108].

*Astragalus membranaceus* has been used traditionally for chronic fatigue, diabetes, ulcers, and as an adjuvant in cancer therapy. Its major bioactive constituents, including APS, flavonoids, and saponins confer broad anti-inflammatory, antioxidant, immunomodulatory, hypoglycemic, and neuroprotective effects. Recent mechanistic analyses emphasize that AS-IV and APS mitigate neuroinflammation, oxidative stress, neuronal apoptosis, and ferroptosis, regulate autophagy, protect the blood–brain barrier, and modulate signalling pathways such as PI3K/Akt, Nrf2, and PPARγ, indicating a multi-target neuroprotective potential [77,109,110].

Preclinical studies demonstrate that APS improve cognitive function, reduce amyloid-β accumulation, and suppress neuroinflammation in experimental models of Alzheimer’s disease, while AS-IV enhances synaptic plasticity, attenuates tau-related and inflammatory pathology, and protects against cerebral ischemia–reperfusion injury [111,112,113]. Furthermore, increasing clinical and translational research interest has emerged regarding *Astragalus membranaceus* as a potential adjunctive therapy for Alzheimer’s disease; however, conclusive clinical evidence remains limited. Variability among *Astragalus* species and the structural complexity of polysaccharides continue to pose challenges for standardization and translational research.

### 2.5. Synergistic Effects of Different Herbal Extracts Combined with Other Therapeutic Agents in Disease Treatment

Combination therapy is a logical strategy for complex, multifactorial disorders because no single agent can simultaneously address the diverse pathological processes that drive chronic diseases. TCM has long promoted multi-component decoctions; modern pharmacology has adopted this concept to explore whether combinations of herbal extracts with conventional drugs can produce synergistic effects that enhance efficacy or reduce toxicity [114,115,116].

Synergy is particularly valuable when disease pathology involves inflammation, oxidative stress, immune dysregulation, and mitochondrial dysfunction, because these processes often interact through shared signalling pathways such as NF-κB, PI3K/Akt, and AMPK. This concept has been studied in cancer and metabolic diseases, where Chinese herbal medicines and related natural products may support conventional therapy by modulating inflammatory pathways, mitochondrial function, oxidative stress, and the tumour microenvironment [117,118,119]. These findings suggest that *Astragalus*-based combinations may contribute to therapeutic benefit through multi-target regulation and immune modulation; however, herb–drug interactions must be considered, and rigorous clinical trials are required to confirm safety and benefit.

*Cordyceps* preparations have been used as nephroprotective adjuvants to cyclosporin-based immunosuppression, and their combination improves renal function by reducing serum creatinine and blood urea nitrogen while mitigating microinflammatory states without compromising immunosuppression. These observations illustrate the capacity of herbal extracts to complement standard treatments while reducing adverse effects [120,121].

Synergy with anti-inflammatory drugs represents another important therapeutic avenue. In a septic rat model, APS combined with ibuprofen reduced plasma TNF-α and IL-6 levels more effectively than either agent alone, and increased expression of the α7 nicotinic acetylcholine receptor, implicating activation of the cholinergic anti-inflammatory pathway. Both *Astragalus* and *Cordyceps* down-regulate NF-κB signalling and NLRP3 inflammasome activation, leading to reduced production of pro-inflammatory cytokines [122,123]. By converging on shared inflammatory pathways, herbal extracts may potentiate the effects of non-steroidal anti-inflammatory drugs or corticosteroids while enabling dose reduction.

Metabolic and cardiovascular diseases are closely associated with oxidative stress and mitochondrial dysfunction. Meta-analyses indicate that *Astragalus* combined with renin–angiotensin–aldosterone system (RAAS) blockers improves therapeutic efficacy in diabetic nephropathy, significantly reducing urinary protein excretion, serum creatinine and blood urea nitrogen compared with RAAS blockade alone. These benefits arise from complementary mechanisms, whereby RAAS inhibitors reduce intraglomerular pressure while *Astragalus* exerts anti-inflammatory and antioxidant effects through Nrf2-related pathways [124,125]. *Cordyceps*-based formulations have been shown to improve hyperglycaemia, dyslipidaemia and renal dysfunction in diabetic models by down-regulating fibrosis-related proteins and up-regulating renoprotective factors such as Smad7 and Klotho [126].

Synergy with anticancer therapies is particularly notable in non-small-cell lung cancer. *Astragalus* formulations combined with platinum-based chemotherapy increase treatment efficacy and reduce adverse events. Preclinical studies demonstrate that APS enhances the antitumour activity of gemcitabine and adriamycin by promoting apoptosis and ferroptosis while modulating the tumour microenvironment. Combination therapy with APS and metformin suppresses lung adenocarcinoma cell proliferation through synergistic inhibition of the PI3K/Akt pathway and enhanced oxidative stress [123,127]. Although *Cordyceps* derivatives are less extensively studied in cancer combination therapy, their immunomodulatory and metabolic regulatory properties suggest potential for synergistic use with chemotherapy, radiotherapy and immunotherapy.

Representative examples of *Astragalus membranaceus* and *Cordyceps sinensis* extracts combined with conventional therapeutic agents, together with their proposed synergistic mechanisms and levels of evidence, are summarized in Table 2.

Mechanistically, synergistic effects arise from complementary actions at multiple biological levels. *Astragalus* and *Cordyceps* polysaccharides enhance innate and adaptive immune responses by promoting macrophage and microglial phagocytosis, increasing natural killer cell activity and shifting macrophage polarization toward an anti-inflammatory M2 phenotype. Both herbs suppress inflammatory signalling via inhibition of NF-κB, PI3K/Akt and MAPK pathways, while activating antioxidant defences through Nrf2/HO-1 signalling [139,140,141]. Mitochondrial protection is achieved through preservation of membrane potential, enhancement of respiratory chain activity and activation of AMPK-mediated energy sensing. The convergence of NF-κB, PI3K/Akt, AMPK, Nrf2 and ERK/CREB pathways provides a mechanistic framework for understanding how herbal extracts complement the selective actions of conventional drugs [142,143,144].

Despite promising results, herb–drug interactions remain a significant concern. Herbal constituents may alter pharmacokinetics by modulating cytochrome P450 enzymes or drug transporters, potentially affecting drug exposure and safety [145]. Most evidence for synergy derives from in vitro or animal studies, whereas high-quality randomized clinical trials remain limited. Therefore, combination therapy with herbal extracts should be pursued cautiously, with emphasis on dose optimization, pharmacokinetic monitoring and rigorous clinical validation.

### 2.6. Research and Clinical Applications of Herbal Extracts in Aging-Related and Neurodegenerative Diseases-Astragalus and Cordyceps as Case Studies

*Astragalus membranaceus* and *Cordyceps sinensis* are emblematic in TCM because they tonify Qi and support vitality, functions that align with modern concepts of metabolic and immune resilience. Both herbs contain diverse bioactive molecules including polysaccharides, saponins, flavonoids and nucleoside derivatives that act on multiple targets involved in inflammation, oxidative stress, mitochondrial function and neuronal survival. Their breadth of activity has made them focal points in research on aging-related chronic diseases and neurodegenerative disorders [146,147,148]. While preclinical studies demonstrate antioxidant, anti-inflammatory and neuroprotective effects, translation into clinical practice remains limited and the mechanisms are still being elucidated.

- Astragalus (*Astragalus membranaceus*)

More than two hundred compounds have been identified in *A. membranaceus*, with three classes dominating its pharmacological profile: polysaccharides (notably *Astragalus* polysaccharides, APS), flavonoids (including isoflavones such as calycosin and formononetin) and triterpenoid saponins (particularly astragalosides such as AS-IV) [18]. APS comprises heteropolysaccharides with antioxidant and immunomodulatory properties; astragalosides are cycloartane-type saponins that modulate membrane fluidity and signal transduction; and flavonoids contribute to free radical scavenging and estrogen-like activity. APS and AS-IV activate Nrf2 and up-regulate antioxidant enzymes (SOD, CAT, GSH-Px) while suppressing NF-κB–dependent pro-inflammatory cytokines (TNF-α, IL-1β, IL-6) and the NLRP3 inflammasome. Formononetin and calycosin further enhance antioxidant defences by activating Nrf2 and protecting against oxidative damage [149,150,151,152]. These bioactive constituents collectively endow *Astragalus* with potent reactive oxygen species (ROS) scavenging capacity, immune enhancement, and anti-inflammatory activity.

Pharmacologically, *Astragalus* demonstrates significant potential for neuroprotection. Roasted *Astragalus* extracts reduce ROS accumulation and lipid peroxidation in neuronal cells by activating the Nrf2/HO-1 pathway and up-regulating the Akt/CREB/BDNF axis, leading to increased brain-derived neurotrophic factor expression and decreased apoptosis markers (Bax, cleaved caspase-3) [141]. AS-IV inhibits NF-κB and MAPK pathways and stabilises mitochondrial membrane potential, thereby preventing β-amyloid-induced mitochondrial permeability transition and neuronal apoptosis. APS also modulates immune responses by shifting microglia and macrophages toward an M2 phenotype and reducing the activation of the NLRP3 inflammasome [94,153,154]. These actions collectively contribute to attenuation of neuroinflammation and support synaptic plasticity, suggesting relevance to aging and neurodegenerative disorders. Preclinical studies provide compelling evidence, but clinical trials remain scarce; thus, the translational potential of *Astragalus* in neurodegeneration is promising yet still uncertain.

- Cordyceps (*Cordyceps sinensis*)

*Cordyceps sinensis* contains a unique set of nucleoside analogues and polysaccharides, the most notable being cordycepin (3′-deoxyadenosine) and cordycepic acid. Cordycepin can act as a purine analogue that modulates adenosine receptors and influences energy metabolism, while cordycepic acid (D-mannitol) contributes to antioxidant and diuretic properties [155]. In addition to these primary constituents, *Cordyceps* produces polysaccharides with immunomodulatory activity and secondary metabolites such as ergosterol and sterol derivatives. Together, these compounds enable *Cordyceps* to regulate the immune system, reduce oxidative stress and inflammation, and support mitochondrial function [156]. For example, cordycepin decreases ROS production and restores mitochondrial membrane potential in neuronal cells exposed to β-amyloid by activating the ERK/CREB pathway. *Cordyceps* preparations also enhance vasodilation by increasing nitric oxide release and inhibiting low-density lipoprotein oxidation, explaining their use in cardiovascular and renal conditions [96,140].

**Figure 3:**
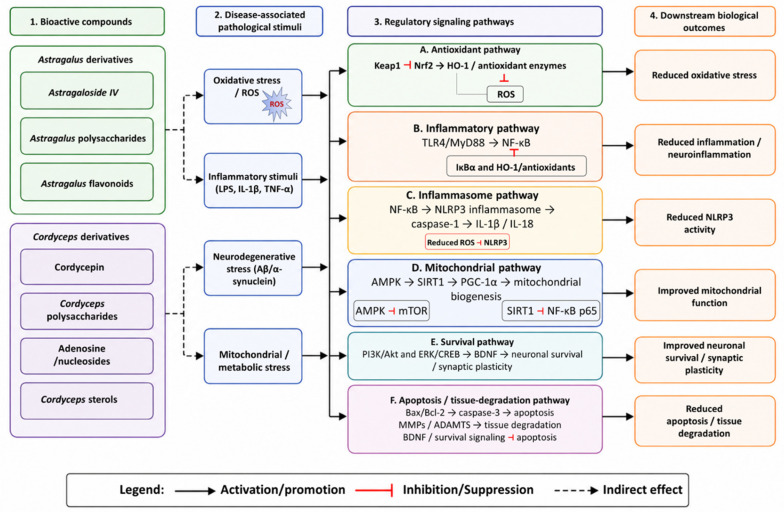
Integrated signaling network regulated by *Astragalus* and *Cordyceps* derivatives in aging-related disorders. The network includes antioxidant, inflammatory, inflammasome, mitochondrial, survival, and apoptosis/tissue-degradation pathways.

Beyond antioxidant and anti-inflammatory actions, *Cordyceps* exhibits notable neuroprotective and anti-aging properties. A recent study demonstrated that cordycepin improves cognitive function in APP/PS1 Alzheimer’s model mice by reprogramming microglial metabolism: it activates hexokinase II and pyruvate dehydrogenase kinase 2, thereby enhancing both glycolysis and oxidative phosphorylation to promote microglial M2 polarization and alleviate mitochondrial damage [120,157]. *Cordyceps* derivatives have been used clinically as adjuncts to immunosuppressive therapy; for example, combining *Cordyceps* with cyclosporin A supports renal function during organ transplantation by reducing nephrotoxicity and modulating immune responses. *Cordyceps militaris* cultivated with *Ginkgo biloba* seeds improves hyperglycaemia and renal dysfunction in diabetic mice, reflecting synergy between *Cordyceps* metabolites and metabolic pathways [120,126]. These findings indicate that cordycepin and other *Cordyceps* compounds can influence metabolic and immune pathways relevant to aging and neurodegeneration; however, clinical evidence remains limited and additional studies are needed to evaluate safety and efficacy. The major signaling pathways through which *Astragalus* and *Cordyceps* exert anti-inflammatory, antioxidant, and neuroprotective effects are summarized in Figure 3.

#### 2.6.1. Cancer

**Figure 4:**
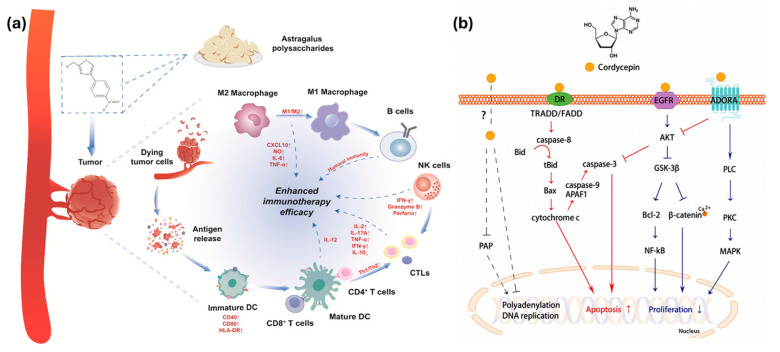
Anticancer mechanisms of Astragalus and Cordyceps derivatives. (**a**) APS-mediated antitumor immunity through dendritic-cell maturation, CD8^+^ T-cell/NK-cell activation, and cytokine remodeling [123]. (**b**) Cordycepin-mediated apoptosis and proliferation control through caspase-related signaling and EGFR/Akt/GSK-3β/cyclin D1 regulation [160]. Adapted with permission from Refs. [123,160].

Cancer is a prototypical complex disease involving aberrant cell proliferation, immune evasion, tumor-promoting inflammation and metabolic reprogramming. *Astragalus* extracts, particularly APS, are widely used as adjuvant therapies in oncology. Meta-analyses and preclinical studies reveal that combining APS with chemotherapy or immunotherapy improves tumour remission rates, reduces tumour volume and prolongs survival while mitigating adverse effects such as neutropenia and neurotoxicity [158,159]. Mechanistically, APS may enhance antitumor immune responses by promoting tumor-cell death, antigen release, dendritic-cell maturation, and antigen presentation, including increased expression of costimulatory molecules such as CD40, CD80, and HLA-DR. This process supports CD8^+^ T-cell activation and may also enhance NK-cell activity through increased IFN-γ, granzyme B, and perforin expression. APS further modulates cytokine profiles by increasing IL-2, IFN-γ, TNF-α, and IL-12, reducing IL-10, and regulating IL-6 in a context-dependent manner. In addition, APS may promote a more antitumor immune microenvironment by influencing macrophage polarization and increasing the M1/M2 macrophage ratio (Figure 4) [122,123]. AS-IV and APS also inhibit NF-κB and PI3K/Akt pathways, promoting apoptosis and ferroptosis when combined with agents like gemcitabine or adriamycin.

In addition, cordycepin may promote apoptosis through death receptor- and caspase-related pathways and suppress cancer-cell proliferation through EGFR/Akt/GSK-3β/cyclin D1-related signaling (Figure 4b) [160]. Recent evidence also suggests that cordycepin and *Cordyceps militaris* extract may sensitize cancer cells and modulate immune responses, supporting their possible role in cancer immunotherapy-related strategies [160,161,162]. However, *Cordyceps* derivatives have not been extensively tested as anticancer adjuvants; their potential may lie in reducing chemotherapy-induced organ toxicity and supporting immune function, but more research is required. Although preliminary clinical evidence suggests benefits in patients with lung, breast, and colorectal cancers, most data derive from small trials or meta-analyses with heterogeneity, and high-quality randomised controlled trials are still needed [123,163].

#### 2.6.2. Diabetes

Diabetes and its complications involve chronic inflammation, oxidative stress and metabolic dysfunction, making them suitable targets for multi-target therapies. *Astragalus* formulations combined with RAAS inhibitors significantly improve outcomes in diabetic nephropathy: co-administration reduces urinary protein excretion, serum creatinine and BUN, and improves glycaemic control compared with RAAS blockade alone. The synergy arises because RAAS inhibitors reduce intraglomerular pressure while *Astragalus* polysaccharides activate Nrf2, suppress NF-κB and enhance antioxidant capacity [124,125,144].

Shenkang injection plus RAAS inhibitors also lowers triglyceride and cholesterol levels and decreases pro-inflammatory cytokines, highlighting a complementary metabolic effect. *Cordyceps militaris* preparations cultivated with *Ginkgo biloba* seeds improve hyperglycaemia, dyslipidaemia and renal dysfunction in type2 diabetic mice by down-regulating fibrosis-related proteins and up-regulating renoprotective proteins such as Smad7 and Klotho [124,126,164,165]. Although these findings suggest that combining *Astragalus* or *Cordyceps* with antidiabetic drugs or RAAS blockers may enhance metabolic control and protect the kidneys, most evidence comes from preclinical or small clinical studies; robust randomised trials are needed to validate efficacy and safety.

#### 2.6.3. Hypertension

Hypertension is tightly linked to vascular inflammation, oxidative stress and RAAS overactivation. *Astragalus* root is traditionally used as a diuretic and Qi-tonifying herb; modern studies indicate that its polysaccharides and flavonoids can moderately lower blood pressure by inhibiting angiotensin-converting enzyme, enhancing nitric oxide release and reducing oxidative stress. Combining *Astragalus* with antidiabetic or statin medications is proposed to provide additional cardiovascular benefits, but pharmacokinetic data suggest that such combinations may prolong drug exposure and necessitate dose adjustment [166]. Shenkang injection plus RAAS blockers has shown cardiorenal protection in patients with diabetic nephropathy, an effect that may also translate to essential hypertension; however, direct clinical evidence in hypertensive patients remains sparse [124]. *Cordyceps* produces vasodilatory and hypocholesterolaemic effects by increasing nitric oxide and suppressing low-density lipoprotein oxidation. Meta-analyses of *Cordyceps* preparations combined with Western medicines for renal dysfunction report improvements in serum creatinine and BUN and suggest that *Cordyceps* helps restore microinflammatory balance [96,120,167]. Although these findings hint at a role for *Astragalus* and *Cordyceps* in hypertension management and their potential synergy with antihypertensive drugs, high-quality clinical trials are lacking and caution is warranted regarding herb–drug interactions.

#### 2.6.4. Osteoarthritis

Osteoarthritis is characterized by inflammatory degradation of articular cartilage and extracellular matrix. In vitro studies using human chondrocyte HTB-94 cells showed that a hydroalcoholic extract of *A. membranaceus* significantly reduced TNF-α-induced expression and secretion of pro-inflammatory mediators (IL-6, IL-1β, IL-8) and matrix-degrading enzymes (MMP-3, MMP-13, ADAMTS-5) without affecting basal levels. These results suggest that *Astragalus* extract can counteract inflammatory signalling and matrix degradation in chondrocytes, supporting its potential as a nutraceutical for joint health [168,169,170].

The mechanisms likely involve inhibition of NF-κB and activation of Nrf2 pathways, consistent with other tissues, though this remains speculative in cartilage. *Astragalus*-derived compounds such as formononetin and astragalin have been shown to inhibit IL-1β-induced inflammatory mediator production in chondrocytes through NF-κB and MAPK suppression [171,172]. *Cordyceps* has not been well studied in osteoarthritis; its anti-inflammatory and antioxidant properties could theoretically synergize with conventional anti-arthritic agents, but empirical evidence is currently minimal.

#### 2.6.5. Parkinson’s Disease

Parkinson’s disease (PD) involves dopaminergic neuron loss, mitochondrial dysfunction and chronic neuroinflammation. Preclinical studies indicate that *Astragalus* saponins and polysaccharides attenuate neurotoxicity in PD models by reducing ROS, inhibiting NF-κB and MAPK signalling and activating Nrf2, thereby preserving mitochondrial integrity [173,174]. AS-IV modulates the Bax/Bcl-2 ratio, preventing neuronal apoptosis, and down-regulates microglial activation via NLRP3 inflammasome suppression. These effects translate to improved motor performance and dopaminergic neuron survival in animal models, but clinical studies are lacking. Cordycepin’s ability to reprogram microglial metabolism, enhance glycolysis and oxidative phosphorylation, and promote M2 polarization suggests potential for mitigating neuroinflammation in PD, though direct evidence in PD models is limited. Overall, both herbs offer promising neuroprotective mechanisms, yet their clinical efficacy in PD remains to be established [173,175,176,177].

#### 2.6.6. Alzheimer’s Disease

Alzheimer’s disease (AD) is characterised by β-amyloid aggregation, tau pathology, synaptic loss and neuroinflammation. Roasted *Astragalus* extracts protect hippocampal neurons by activating Nrf2/HO-1 and Akt/CREB/BDNF pathways, reducing ROS and increasing BDNF levels, while decreasing pro-apoptotic proteins such as Bax and cleaved caspase-3 [141]. AS-IV prevents β-amyloid-induced mitochondrial permeability transition, reduces ROS production and improves cognitive behaviour in AD models [153,178]. More broadly, nutraceutical-based strategies have also been investigated for their ability to modulate β-amyloid aggregation and downstream neurotoxic pathways in Alzheimer’s disease, supporting the therapeutic relevance of multi-target natural compounds in neurodegenerative disorders [179].

APS inhibits microglial overactivation and NLRP3 inflammasome assembly, promotes M2 polarization and maintains blood–brain barrier integrity through PPARγ and Nrf2 pathways. Cordycepin significantly improves cognitive function in APP/PS1 mice by promoting metabolic reprogramming of microglia; activation of hexokinase II and pyruvate dehydrogenase kinase 2 enhances glycolysis and oxidative phosphorylation, shifting microglia toward an anti-inflammatory phenotype and mitigating mitochondrial damage [157,180]. The mechanistic evidence and evidence levels of Astragalus membranaceus and Cordyceps sinensis across disease models are summarized in Table 3.

## 3. Challenges and Limitations

Both *Astragalus* and *Cordyceps* species show marked variability in chemical composition depending on geographic origin, cultivation conditions and processing. A metabolomics study on *Astragalus membranaceus* roots found that age and origin significantly affected saponin and flavonoid profiles; AS-IV, commonly used as a quality marker, varied widely among samples, and older roots contained more flavonoids while saponins and isoflavonoids distinguished geographical origin [195]. Another study reported that Radix Astragali from Gansu province had lower active ingredients (astragaloside and calycosin) but higher yield compared with daodi (authentic) regions, emphasising the need for multi-index quality control. Adulteration with other *Astragalus* species further complicates quality assurance [195,196].

For *Cordyceps*, authentic *Ophiocordyceps sinensis* is rare and expensive; many preparations use cultured mycelia or related species (e.g., *Cordyceps militaris*), leading to variable levels of cordycepin, polysaccharides and peptides. Differences in fermentation conditions, substrates and harvest times affect bioactive content, yet these factors are seldom reported in clinical studies.

### 3.1. Standardization and Quality Control Challenges

Quality control typically relies on marker compounds (AS-IV for *Astragalus* and cordycepin for *Cordyceps*), but single markers do not capture the multi-component nature of the extracts. Fingerprinting methods using chromatographic or spectrometric profiles have been proposed [196], but batch-to-batch consistency remains problematic. The metabolomics study noted that different analytical techniques (TLC, HPLC, NMR, MS) yield variable results, underscoring difficulties in defining activity-related markers [195]. Without standardized preparation and reporting, reproducibility of pharmacological and clinical findings is limited.

### 3.2. Pharmacokinetic and Bioavailability Issues

Triterpenoid saponins such as AS-IV have poor intestinal permeability and low lipophilicity, resulting in low oral bioavailability (2–7%) [197,198]. *Astragalus* polysaccharides (APS) are large (10–50 kDa) and hydrophilic; their absorption efficiency is low because of poor intestinal permeability. APS are mainly metabolized by intestinal microbiota and distributed to the liver, kidneys, spleen and lungs. Nanocarrier formulations improve APS solubility and stability but have not been standardised in clinical trials [107]. Cordycepin has extremely short half-life (≈5 min) and is rapidly excreted; oral dosing yields minimal systemic exposure [199]. These pharmacokinetic challenges complicate dose determination, affect interactions with other drugs and may lead to unpredictable efficacy.

More importantly, these pharmacokinetic limitations also affect the interpretation of the mechanistic evidence discussed above. Although activation of Nrf2/HO-1, inhibition of NF-κB/NLRP3 signaling, modulation of AMPK/mTOR and PI3K/Akt pathways, and suppression of apoptosis are frequently reported in cell-based studies [142], these effects may not occur with the same intensity in vivo if active constituents do not reach sufficient concentrations in target tissues. Conversely, some in vivo effects may arise indirectly through gut microbiota remodeling, immune modulation, altered drug metabolism, or bioactive metabolites rather than direct action of the parent compounds [70]. Therefore, future studies should combine pharmacokinetic measurements with pathway analysis by identifying circulating metabolites, measuring tissue distribution, evaluating blood–brain barrier penetration where relevant, and correlating pathway modulation with exposure parameters such as Cmax, half-life, and area under the curve [70,142,200].

### 3.3. Limitations of Current Clinical Studies

Clinical evidence for *Astragalus* and *Cordyceps* derivatives in aging-related chronic and neurodegenerative diseases is limited. A 2025 review emphasised that current evidence is dominated by in vitro and animal studies; existing human trials have small sample sizes, short durations and inadequate controls, and long-term safety particularly at high doses or in vulnerable populations remains unclear [110]. In systematic reviews of *Ophiocordyceps* preparations for Hashimoto’s thyroiditis, most included studies were small, single-center trials with sample sizes of 47–120 participants; many lacked proper randomisation, blinding and protocol registration, and adverse events were rarely reported [201]. Meta-analyses of Bailing capsules for nephrotic syndrome highlight poor methodological quality, high heterogeneity due to differences in patient characteristics, treatment doses and duration, and small sample sizes, making it difficult to assess long-term safety [202]. Overall, the heterogeneity of formulations and the low quality of trials hamper evidence synthesis and translation to clinical practice.

### 3.4. Safety Profiles, Potential Toxicities, and Herb–Drug Interactions

Safety evaluation is a necessary extension of efficacy assessment for *Astragalus* and *Cordyceps* derivatives, particularly because the target populations often include older adults with chronic diseases, comorbidities, and polypharmacy. Although both herbs have long histories of use as traditional tonic medicines, historical use alone is insufficient to establish clinical safety, especially when preparations differ in species origin, extraction method, dose, treatment duration, route of administration, and chemical standardization. This is particularly relevant because *Astragalus* and *Cordyceps* products vary substantially in their polysaccharide, saponin, flavonoid, nucleoside, sterol, and other metabolite profiles, which may influence pharmacological activity, toxicity, and interaction risk [112,203,204].

For *Astragalus* derivatives, polysaccharide-rich preparations are commonly reported to have relatively low toxicity in experimental and clinical contexts; however, robust long-term safety data remain limited, and adverse-event monitoring is not always systematic across studies [205]. Mild gastrointestinal symptoms, including nausea, abdominal discomfort, or diarrhea, may occur with some herbal preparations, particularly at high doses or when extract composition is not well standardized [206]. *Astragalus* saponins, including AS-IV, should also be considered carefully because saponins possess amphiphilic structures that can interact with biological membranes; therefore, high exposure or poorly characterized extracts may increase the risk of nonspecific irritant or membrane-related effects, although direct clinical evidence for such toxicity remains limited. In addition, *Astragalus* has immunomodulatory activity, which may be beneficial in inflammatory, metabolic, or cancer-related contexts, but it also supports caution in patients with autoimmune diseases or in those receiving immunosuppressive therapy, where unintended pharmacodynamic interactions are possible [207,208].

*Cordyceps* derivatives present a distinct safety profile because their major bioactive compounds include nucleosides, polysaccharides, sterols, and other fungal metabolites. *Cordycepin*, a 3′-deoxyadenosine nucleoside analogue, has been linked to effects on adenosine-related signaling, RNA metabolism, and energy-regulatory pathways; these mechanisms support its therapeutic potential but also suggest that high systemic exposure, metabolic stabilization, or coadministration with agents affecting nucleoside metabolism could increase safety concerns [87,209]. *Cordyceps* preparations are also highly heterogeneous, and differences among wild *Ophiocordyceps sinensis*, cultured mycelial products, and *Cordyceps militaris* preparations may produce variable chemical composition and biological responses [96]. In renal and transplant-related settings, *Cordyceps* products have been investigated as adjuncts to conventional therapy, but systematic reviews indicate that adverse-event reporting has often been incomplete or poorly standardized; therefore, claims of safety should be interpreted cautiously rather than as proof of absence of risk [210,211,212].

Herb–drug interactions are clinically relevant but remain insufficiently characterized for *Astragalus* and *Cordyceps* derivatives. Potential herb–drug interactions require particular attention in aging-related diseases because patients with diabetes, cardiovascular disease, cancer, osteoarthritis, Alzheimer’s disease, or Parkinson’s disease often receive multiple conventional medications [5,213]. Several therapeutic combination contexts have been reported, including *Astragalus* with RAAS inhibitors in diabetic nephropathy, *Astragalus*-based preparations with chemotherapy, and *Cordyceps* with cyclosporine-related immunosuppressive therapy; however, most studies evaluated efficacy rather than detailed pharmacokinetic interactions or safety outcomes. Mechanistically, interaction risk is plausible because some herbal constituents may affect drug metabolism, phase II conjugation, transporter activity, or immune responses [125,214]. Therefore, future studies should specifically examine drug exposure, adverse events, dose adjustment, and safety monitoring when *Astragalus* or *Cordyceps* derivatives are used with conventional therapies.

In addition to general toxicity and herb–drug interaction concerns, specific physiological effects should be considered in vulnerable patients. Some Astragalus-derived preparations may influence vascular function and blood-pressure regulation; therefore, caution is required when they are used with antihypertensive drugs, because additive blood-pressure-lowering effects cannot be excluded [215,216]. Effects on blood rheology and coagulation should also be considered. *Cordyceps militaris* extracts and cordycepin-enriched preparations have been reported to show antiplatelet and antithrombotic activities in experimental systems, suggesting possible effects on platelet aggregation and thrombus formation [217]. These properties may be beneficial in cardiovascular contexts but may also increase safety concerns in patients receiving anticoagulant or antiplatelet therapy, individuals with bleeding disorders, or patients preparing for surgery. Allergic and immune-related reactions also require attention, particularly in patients with allergic tendencies, autoimmune diseases, or immunosuppressive therapy [218,219]. Therefore, future clinical studies should include blood-pressure monitoring, coagulation-related assessment, allergic reaction reporting, and careful documentation of concomitant medication use.

Product quality and standardization also represent major safety considerations. Batch-to-batch variability, species misidentification, cultivation conditions, extraction procedures, contamination, and adulteration may substantially alter the composition and safety profile of *Astragalus* and *Cordyceps* products [204,211,220]. Therefore, future investigations should incorporate chemical fingerprinting, marker-compound quantification, contaminant screening, and transparent reporting of extraction and manufacturing conditions. Clinical studies should also include systematic adverse-event monitoring, liver and kidney function assessment, dose–response evaluation, medication-history documentation, and post-treatment follow-up. Overall, Astragalus and Cordyceps derivatives represent promising adjunctive therapeutic candidates, but their safety profiles require continued rigorous evaluation before broad clinical implementation [210,221,222].

## 4. Discussion

### 4.1. Integrated Comparison of Astragalus and Cordyceps

Both *Astragalus membranaceus* and *Ophiocordyceps sinensis* possess multi-target mechanisms relevant to aging-related chronic diseases and neurodegeneration. *Astragalus* derivatives particularly AS-IV, calycosin and APS exert antioxidant, anti-inflammatory, immunomodulatory and metabolic effects via Nrf2, NF-κB, PPAR-γ and PI3K/AKT/mTOR pathways. APS regulate gut microbiota and promote insulin sensitivity and neuronal survival [107,223]. *Cordyceps* derivatives cordycepin, polysaccharides and peptides modulate energy metabolism, enhance mitochondrial function, suppress inflammation and improve renal and immune function. Cordycepin’s ability to cross the blood–brain barrier [224,225], suggests direct neuroprotective potential, whereas polysaccharide–peptide complexes stimulate antioxidant and immunoregulatory pathways.

Distinct mechanistic strengths arise from these compositions: *Astragalus* saponins target lipid metabolism and vascular inflammation, which is reflected in synergistic effects with statins in atherosclerosis models [226]. APS influence insulin signalling and microglial activation, making them attractive for metabolic and neurodegenerative conditions. *Cordyceps* nucleosides influence adenosine receptors and immune checkpoints; combination with renin–angiotensin system inhibitors improves renal hemodynamics and reduces proteinuria [227]. Cordycepin’s rapid metabolism, however, demands delivery systems or co-administration with adenosine deaminase inhibitors to maintain therapeutic levels.

### 4.2. Shared and Disease-Specific Signaling Pathways

The Nrf2/HO-1, NF-κB/NLRP3, AMPK–mitochondrial, PI3K/Akt/mTOR, PPARγ, and CREB/BDNF pathways were emphasized because they represent shared signaling nodes across oxidative stress, inflammation, metabolic imbalance, mitochondrial dysfunction, blood–brain barrier regulation, synaptic plasticity, and neuronal survival [18,190,228]. However, their inclusion should be interpreted as a framework for organizing mechanistic evidence, not as proof that all pathways are equally validated across all diseases.

The strength of evidence differs across compounds and disease contexts. Nrf2/HO-1 and NF-κB/NLRP3 have relatively strong preclinical support, especially for Astragalus polysaccharides and AS-IV. In contrast, cordycepin-related regulation of AMPK–mitochondrial function, microglial metabolism, and CREB/BDNF signaling is better supported in Alzheimer’s disease models than in Parkinson’s disease models, where disease-specific validation remains limited. Similarly, *Cordyceps* evidence in osteoarthritis remains comparatively weak and is mostly inferred from broader antioxidant and anti-inflammatory activity rather than strong OA-specific pathway validation [229,230,231].

These pathway findings should also be interpreted according to disease context. PI3K/Akt/mTOR is a useful example because it may support neuronal survival and autophagy regulation in neurodegenerative or ischemic models, but in cancer the same pathway may contribute to cell survival, proliferation, metastasis, and therapy resistance [232,233]. Therefore, future studies should connect pathway modulation with disease-specific functional outcomes instead of relying only on broad antioxidant or anti-inflammatory markers.

### 4.3. Relevance of Multi-Target Pharmacology to Aging Biology

Aging is characterised by interconnected processes oxidative stress, mitochondrial dysfunction, chronic inflammation, impaired autophagy and metabolic dysregulation that contribute to cardiovascular, metabolic and neurodegenerative diseases. Single-target drugs often fail to address this complexity. Multi-target agents such as *Astragalus* and *Cordyceps* derivatives act concurrently on antioxidant, anti-inflammatory, metabolic and immune pathways, offering a systems-level approach to delay or mitigate age-related pathologies. For example, AS-IV combined with atorvastatin synergistically modulated lipid profiles and inflammatory cytokines via complementary pathways, and *Ophiocordyceps* preparations added to ACE inhibitors improved both renal function and glycaemic control [226,234,235,236]. Such evidence suggests that multi-target herbal medicines can enhance the efficacy of conventional therapies while potentially reducing required doses and side-effects.

### 4.4. Translational Implications and Cautions

The preclinical and limited clinical data reviewed here support the realistic positioning of *Astragalus* and *Cordyceps* derivatives as adjuncts rather than standalone cures. Their complex pharmacology may complement existing drugs in managing dyslipidaemia, hypertension, renal dysfunction and neurodegenerative symptoms. However, translation into evidence-based practice faces several hurdles: poor bioavailability of saponins and nucleosides, variability of herbal materials, lack of standardized formulations, and inadequate clinical trial quality.

The limited oral bioavailability and short biological half-life of several active constituents restrict the extent to which efficacy can be claimed for crude or non-standardized raw materials [237]. For example, many flavonoids undergo extensive phase II metabolism and transporter-mediated efflux, polysaccharides are poorly absorbed as intact macromolecules and may act indirectly through gut microbiota-derived metabolites, and cordycepin is rapidly metabolized by adenosine deaminase [238,239]. Therefore, evidence obtained using purified compounds, high in vitro concentrations, injectable formulations, or optimized extracts cannot be directly extrapolated to ordinary dietary supplements or crude herbal powders. The current evidence supports pharmacological potential rather than confirmed clinical effectiveness. Stronger efficacy claims require standardized preparations, validated marker-compound content, pharmacokinetic data, dose–response studies, and controlled clinical trials [240,241,242,243].

Herb–drug interactions and pharmacokinetic uncertainty necessitate cautious co-administration and close monitoring [107,244]. The predominance of small, single-center trials with short follow-up and high risk of bias limits confidence in reported benefits [201]. These translational issues indicate that future research should move beyond broad efficacy claims and adopt more specific strategies for standardization, mechanistic validation, pharmacokinetic optimization, and disease-specific clinical evaluation, as outlined in the Conclusion.

### 4.5. Standardization, Exposure, and Biological Plausibility

The variability of Astragalus and Cordyceps-derived materials represents a major limitation for interpreting reported immunomodulatory and antitumor effects. Concentrations of putative active constituents can differ according to species, geographic origin, cultivation conditions, harvest time, postharvest processing, extraction solvent, extraction yield, and storage [99,245,246]. Consequently, pharmacological effects observed with purified compounds or chemically enriched extracts may not be reliably reproduced after ingestion of crude raw materials, particularly if systemic or local tissue concentrations remain below biologically active levels [247].

This issue is especially relevant for antitumor and immune-modulating effects, which are often demonstrated in vitro using concentrations that may exceed achievable plasma or tissue exposure after oral administration [248]. Therefore, the biological plausibility of these effects should be evaluated using pharmacokinetic and pharmacodynamic evidence [248]. For relatively small molecules, including cordycepin, adenosine-related nucleosides, flavonoids, and saponins or their metabolites, plasma concentration, half-life, metabolism, tissue distribution, and active metabolite formation are critical [249,250].

Future studies should report marker-compound concentrations, batch-to-batch variability, extraction yield, bioavailability, achievable plasma or tissue exposure, and exposure–response relationships to improve translational interpretation and reproducibility [251,252]. In the absence of such pharmacokinetic and standardization data, the reported immune-modulating and antitumor effects of orally consumed crude preparations should be interpreted cautiously and may be more appropriately considered indicative of mechanistic or preclinical potential rather than established in vivo efficacy.

## 5. Conclusions

Aging-related chronic diseases and neurodegenerative disorders are driven by complex and interdependent pathological processes, including oxidative stress, chronic inflammation, mitochondrial dysfunction, metabolic disturbance, and immune imbalance. Such biological complexity limits the effectiveness of single-target therapeutic strategies and highlights the need for interventions capable of modulating multiple disease-relevant pathways simultaneously. Within this context, traditional Chinese herbal medicines provide a valuable systems-oriented framework, and *Astragalus membranaceus* and *Cordyceps* species emerge as particularly important examples because of their long history of use, chemical diversity, and growing modern pharmacological support.

The evidence reviewed here indicates that *Astragalus*- and *Cordyceps*-derived compounds, especially polysaccharides, astragalosides, flavonoids, and cordycepin, can influence a wide range of mechanisms relevant to aging biology and degenerative disease, including NF-κB, PI3K/Akt, AMPK, Nrf2, mitochondrial bioenergetics, and inflammatory immune signaling. Across preclinical models, these herbs have shown promising effects in metabolic disorders, cardiovascular dysfunction, osteoarthritis, cancer, and neurodegenerative diseases such as Alzheimer’s and Parkinson’s disease. However, the current evidence base remains weighted toward in vitro and animal studies, while human data are still limited by small sample sizes, heterogeneous formulations, adjunctive-study designs, inconsistent endpoints, and insufficient pharmacokinetic characterization.

Currently, *Astragalus* and *Cordyceps* derivatives should be regarded primarily as adjunctive therapies rather than standalone replacements for established treatments, particularly within integrative therapeutic strategies. Future research should therefore move from broad efficacy claims toward specific, testable, and reproducible study designs. First, herbal preparations should be chemically characterized by consistently reporting established quality-control markers and compositional fingerprints. For Astragalus, future studies should quantify key constituents such as AS-IV, APS, calycosin, and formononetin, whereas *Cordyceps* studies should clearly report the species or strain, cultivation conditions, and levels of cordycepin, adenosine, polysaccharides, and sterol derivatives. Second, mechanistic studies should directly test pathway causality rather than only reporting pathway-associated changes. Major pathways discussed in this review, including Nrf2/HO-1, NF-κB/NLRP3, AMPK–mitochondrial signaling, PI3K/Akt/mTOR, PPARγ, and CREB/BDNF, should be validated using inhibitor-based, genetic, or rescue approaches. Third, pharmacokinetic and formulation studies should define plasma and tissue exposure, active metabolites, blood–brain barrier penetration, dose–response relationships, and strategies to improve the low bioavailability, limited systemic exposure, or rapid metabolism of key constituents such as AS-IV and cordycepin. The indirect actions of Astragalus polysaccharides through intestinal degradation and microbiota-mediated immunometabolic effects also require further clarification. Finally, clinical studies should be disease-specific and should include predefined endpoints, such as metabolic, cardiovascular, cognitive, motor, inflammatory, oxidative-stress, quality-of-life, and safety outcomes. In summary, Astragalus and Cordyceps derivatives hold therapeutic promise for aging-related chronic and neurodegenerative diseases, but their translation into safe and clinically meaningful practice will require standardized chemistry, pathway-specific validation, pharmacokinetic support, and indication-specific clinical evaluation.

## Figures and Tables

**Table 1:** Extraction techniques for major bioactive compound classes in *Astragalus* and *Cordyceps*.

Compound Class	Key Structural Features	Major Biological Role	Typical Extraction Method	References
Saponins	Triterpenoid or steroid aglycone linked to sugar moieties	Anti-inflammatory, immunomodulatory, metabolic regulation	Hot-water or aqueous ethanol extraction	[83]
Polysaccharides	High-molecular-weight carbohydrate polymers	Immunomodulation, antioxidant activity	Hot-water extraction; enzyme-assisted extraction	[84,85]
Flavonoids	Polyphenolic C6-C3-C6 structure	Antioxidant and anti-inflammatory effects	Hydroalcoholic solvent extraction	[86]
Nucleosides (e.g., cordycepin)	Adenosine derivatives lacking 3′-OH group	Neuroprotective and metabolic regulation	Ultrasound-assisted or hydroalcoholic extraction	[87]
Organic acids	Small polar molecules with carboxyl groups	Antioxidant and metabolic modulation	Water or mild solvent extraction	[88]
Lipophilic alkaloids/sterols	Hydrophobic heterocyclic or steroid structures	Membrane and signaling modulation	Non-polar solvents or supercritical CO_2_ extraction	[89]

**Table 2:** Synergistic Effects of *Astragalus membranaceus* and *Cordyceps sinensis* Extracts Combined with Conventional Therapies.

Herbal Extract	Co-Administered Drug or Therapy	Disease Indication	Proposed Synergistic Mechanism	Level of Evidence
*Astragalus membranaceus* polysaccharides (APS)	Voriconazole	Antifungal therapy	Regulates voriconazole metabolism, potentially mitigating hepatotoxic effects [128].	In vitro
*Astragalus membranaceus* (CHM formulas)	Platinum-based chemotherapy (e.g., cisplatin)	Advanced non-small cell lung cancer (NSCLC)	Increased tumor response rate; reduced chemotherapy-induced toxicity, including nausea, vomiting, and myelosuppression [129].	Phase II clinical trial
*Astragalus membranaceus* (CHM formulas)	Chemotherapy (CT)	Cervical cancer	Increased tumor response rate (CR/PR); improved Karnofsky performance status; reduced CT-induced toxicity (nausea/vomiting, alopecia, neurotoxicity, hepatic and renal toxicity) [130].	Systematic review and meta-analysis of RCTs
AS-IV	Cisplatin	Non-small cell lung cancer (NSCLC)	Sensitizes cancer cells to cisplatin through modulation of intracellular signaling pathways [131].	Animal model/In vitro
*Cordyceps sinensis* extract/Cordycepin	Cisplatin	Non-small cell lung cancer (NSCLC)	Synergistic anti-proliferative effects; reversal of cisplatin resistance via AMPK activation and AKT inhibition [132,133].	Animal model/In vitro
*Astragalus membranaceus* extract (Axtragyl^®^)	Not applicable	Osteoarthritis (OA)	Anti-inflammatory and chondroprotective effects mediated by inhibition of NF-κB signaling, matrix metalloproteinases (MMPs), and pro-inflammatory cytokines [134].	Human randomized controlled trial
*Cordyceps sinensis*/Cordycepin	Interleukin-1β (IL-1β)	Osteoarthritis (OA)	Suppresses inflammatory responses by inhibiting NF-κB pathway activation in chondrocytes [135,136].	In vitro
*Astragalus membranaceus* injection	Conventional heart failure therapy	Chronic heart failure	Activates mitophagy and preserves mitochondrial function through inhibition of the AKT/mTOR pathway [137].	Animal model
*Astragalus membranaceus* (CHM formulas)	Paclitaxel	Advanced NSCLC	Alleviates cancer-related fatigue and improves systemic inflammatory status [138].	Clinical report
*Astragalus membranaceus* (CHM formulas)	Immune checkpoint inhibitors	Advanced solid cancers	Enhances immunotherapy responsiveness via PD-1/PD-L1 axis modulation and gut microbiota regulation. Chinese Clinical Trial Registry; 2023. Registration No.: ChiCTR2300068896. Available from: https://www.chictr.org.cn/showprojEN.html?proj=189330 (accessed on 10 April 2026).	Phase IV randomized clinical trial (registered; results pending)

**Table 3:** Mechanistic Evidence and Evidence Levels of *Astragalus membranaceus* and *Cordyceps sinensis* Across Disease Models.

Disease	Herbal Extract	Key Bioactive Compounds	Mechanistic Targets	Evidence Level
Cancer	*Astragalus membranaceus*	AS-IV, Polysaccharides (APS)	Immune modulation (↑ NK cell activity), PD-L1 inhibition, NF-κB pathway inhibition, apoptosis induction	Preclinical (cell/animal models) [130,181]; Clinical (Phase I/II trials) [182]
Cancer	*Cordyceps sinensis*	Cordycepin	Immune modulation (↓ PD-L1 expression), reversal of cisplatin resistance (AMPK activation, AKT inhibition), apoptosis induction	Preclinical (cell/animal models) [132,181]
Diabetes/Diabetic Kidney Disease (DKD)	*Astragalus membranaceus*	AS-IV, Polysaccharides (APS)	Anti-inflammatory (↓ NF-κB), antioxidant activity, podocyte protection, anti-fibrotic effects	Preclinical (cell/animal models) [183]; Clinical (observational/Phase IV) [184,185]
Diabetes/Diabetic Kidney Disease (DKD)	*Cordyceps sinensis*	Cordycepin, Bailing capsule (formulation)	Anti-diabetic effects, prevention of metabolic syndrome, renoprotection in animal models	Preclinical (animal models) [186]; Clinical (systematic reviews) [187]
Hypertension	*Astragalus membranaceus*	Polysaccharides (APS), flavonoids	Vasodilation (NO-dependent), anti-inflammatory and antioxidant effects, calcium channel modulation	Preclinical (animal models); Clinical (observational/pilot trials) [188,189].
Osteoarthritis (OA)	*Astragalus membranaceus*	Polysaccharides (APS), AS-IV	NF-κB inhibition, ↓ MMP-3/13, ↓ pro-inflammatory cytokines (TNF-α, IL-1β, IL-6), chondroprotection	Preclinical (cell/animal models) [134,170]; Clinical (RCT) [134]
Osteoarthritis (OA)	*Cordyceps sinensis*	Cordycepin	NF-κB inhibition, ↓ MMP-13, ↓ inflammatory mediators (NO, PGE_2_)	Preclinical (cell models) [135,136]
Parkinson’s Disease (PD)	*Astragalus membranaceus*	Polysaccharides (APS)	Mitochondrial protection (↑ CEND1), ↓ oxidative stress, ↓ neuroinflammation, neuronal stabilization	Preclinical (animal models) [190].
Parkinson’s Disease (PD)	*Cordyceps sinensis*	Cordycepin	Mitochondrial protection (↓ mitochondrial ROS), protection of dopaminergic neurons	Preclinical (cell/animal models) [191,192]
Alzheimer’s Disease (AD)	*Astragalus membranaceus*	Roasted extract, Polysaccharides (APS)	Modulation of β-amyloid pathology, mitigation of neuroinflammation, oxidative stress reduction (Nrf2 pathway)	Preclinical (animal models) [141,193]
Alzheimer’s Disease (AD)	*Cordyceps sinensis*	Cordycepin	Neuroprotection against Aβ-induced apoptosis, delay of cellular senescence, mitochondrial support	Preclinical (cell/animal models) [140,194].

↑ = Increase; ↓ = decrease.

## Data Availability

No new data were created or analyzed in this study. Data sharing is not applicable to this article.
